# Characterizing the presence of polychlorinated biphenyls within Biota Found in New Mexico

**DOI:** 10.1371/journal.pone.0357904

**Published:** 2026-09-09

**Authors:** Justin Clements, Jenna E. Stanek, Kylie Gallegos, Jessica Celmer, Jesse Berryhill, Andrew Thiros, Mark A. Peyton, Shannon M. Gaukler

**Affiliations:** 1 Environmental Protection and Compliance Division, Environmental Stewardship Group, Los Alamos National Laboratory, Los Alamos, New Mexico, United States of America; 2 H3 Environmental, LLC, Albuquerque, New Mexico, United States of America; 3 Office of General Counsel Division, Intellectual Property, Environmental, and Regulatory Group, Los Alamos National Laboratory, Los Alamos, New Mexico, United States of America; 4 Department of Biology, New Mexico State University, Las Cruces, New Mexico, United States of America; University of Arkansas for Medical Sciences College of Pharmacy, UNITED STATES OF AMERICA

## Abstract

Polychlorinated biphenyls (PCBs) are a group of industrial chemicals that were widely used in the United States before being banned by the Toxic Substances Control Act in 1976. Concerns regarding PCBs arose in the 1960s due to their toxicity, classification as a carcinogenic compound, and longevity in the environment. Although the production of PCBs in the United States is currently banned, PCBs can still be found in legacy equipment, and the environment and PCBs are still unintentionally produced in common building materials. To document the concentration of PCBs in biota predominantly found in northern New Mexico, biota (mammalian, avian, and reptilian) were opportunistically collected from 2009 to 2025. We grouped biota species into feeding guilds (carnivore, herbivore, and omnivore) to examine whether different feeding guilds had higher or lower proportions of total PCBs, PCB homologs, dioxin-like PCB congeners, and toxic equivalents. When we examined the concentrations of PCB homologs and total PCBs, we found the highest abundance of PCBs in the carnivore feeding guild compared with both herbivores and omnivores. We also observed differences of dioxin-like PCB congeners among feeding guilds, examined in this study. Toxic equivalents were also observed at higher values in carnivores. Furthermore, we demonstrate the biomagnification of PCBs across trophic levels, with higher concentrations observed in mountain lions relative to their primary ungulate prey (mule deer and elk). A comparison of biota revealed a significant difference in concentrations of PCBs found in samples (mule deer and elk) collected in developed areas when compared with rural locations, with the developed areas having higher concentrations. Our investigation provides significant insight into the distribution, composition, and biomagnification of PCB in terrestrial animals in New Mexico, including the biomagnification of PCBs within the carnivore feeding guild, which could be at a higher risk of adverse effects from PCB exposure.

## Introduction

Polychlorinated biphenyls (PCBs) are a group of man-made organic compounds that were heavily used in industrial production in the 20th century due to their beneficial chemical properties [1]. PCBs are composed of two phenolic rings attached with a single bond generating a biphenyl compound [2]. Replacement of the hydrogen atoms within the biphenyl group with a chlorine forms PCB homologs/congeners [2]. Within each phenolic ring are five locations in which a chlorine can be substituted for a hydrogen atom. As a result, 10 different PCB homologs (number of chlorines attached to the biphenyl ring, including monochloro, dichloro, trichloro, tetrachloro, pentachloro, hexachloro, heptachloro, octachloro, nanochloro, and decachloro homologs with 209 different congeners (position of the chlorines on the biphenyl ring)) can be generated [2]. Although there is no natural source or production of PCBs in the environment, these compounds were generated in large quantities through industrial processes in which chlorine was added to batches of biphenyls [3]. Chlorine was incorporated until the required percentage in the mixture was met, which usually resulted in a mixture of different homologs. These PCB homologs were produced in the United States for approximately 50 years (1929–1977, banned in 1979) and were predominantly used in heat transfer fluids in transformers and capacitors due to their desirable chemical and thermal properties [4]. This resulted in heavy PCB use in a multitude of industrial processes, including heavy equipment and large electrical transformers, and building materials including caulking. It has been suggested that approximately 1.1 billion pounds of PCBs was produced by the United States [4]. As a result of their industrial use, large quantities of PCBs have been released into the environment through electrical waste, poor handling, spills, and old decaying equipment, including transformers and capacitors [5]. In this study we sought to examine the distribution of PCB concentrations within and among terrestrial biota (mammalian, avian, and reptilian) found in New Mexico, United States, including apex predators.

Concerns regarding PCBs arose in the 1960s due to their toxicity, classification as a carcinogenic compound, and longevity in the environment [2,6]. In 1971, the Toxic Substances Control Act (TSCA) was introduced to the United States Congress and was passed into law in 1976 [7]. This legislation, approved by President Gerald Ford, remains one of the driving forces for the United States Environmental Protection Agency (EPA) to control toxic substances. The act focuses on six chemical substances—PCBs, asbestos, radon, lead, mercury, and formaldehyde—but allows the EPA to evaluate all existing chemicals and impose regulations on any chemicals deemed to pose risk to public safety [7]. The TSCA is heavily focused on PCBs, prohibiting the manufacturing and distribution of these substances and establishing regulations for appropriate disposal [7]. Though PCB production has ceased in the United States, legacy contamination can still be found in many rural, urban, and commercial areas [8–11]. Further, PCBs are currently unintentionally produced as a byproduct of commercial processes, including PCB-11 which has been measured in humans [12,13]. This contamination still leads to health concerns in biota exposed to these legacy chemicals. PCBs in the outdoor environment are typically found in higher concentrations in areas where heavy industrial operations were conducted [14]. PCBs can also be found at higher concentrations in indoor settings, including schools that used PCB in building materials [15]. Some of the most heavily polluted areas in the United States have resulted from dumping of chemicals into waterways by large manufacturing plants and paper mills. One of the most infamous examples includes the dumping of more than 1.3 million pounds of PCBs into the Hudson River from General Electric’s capacitor manufacturing plant in New York, United States [16]. This environmental catastrophe has resulted in a multi-billion-dollar effort to clean the river—an effort that has spanned almost four decades.

PCBs were used in multiple applications in urban environments. The applications can be broken down into open and closed systems. In closed systems (dielectric fluids in capacitors and transformers), PCBs were not expected to be released into the environment [17]. PCBs were also used in open systems (plasticizers, flooring material, inks, pesticide fillers, lubricating oils, metal coatings, and many more applications) in which PCBs could be directly released into the environment [17]. Both applications (leaking of closed systems) led to environmental releases and exposures of PCBs in the environment—especially true in urban areas where the use of these materials was more common.

Living organisms accumulate PCB compounds in their tissues through uptake from soil, water, food, direct contact with their epidermal layer, and inhalation through volatilization [2,15]. After entering an organism, PCBs tend to accumulate in fat bodies and fatty tissue [18]. PCBs are known to be stable and do not tend to break down easily, which can result in high accumulation of PCBs within fats and tissues [19]. In general, less-chlorinated PCB compounds are more readily metabolized and eliminated than their highly chlorinated PCB counterparts, which are more readily stored within fat tissues [20]. Due to their stability and lack of elimination, PCBs tend to bioaccumulate in older animals and biomagnify up the trophic level, which can lead to significantly higher PCB concentrations in top predators. Because of the metabolic and elimination properties of PCBs by biota, the more highly chlorinated compounds are expected to accumulate in higher trophic level animals [21]. In addition, 12 PCB congeners (PCB −77, −81, −105, −114, −118, −123, −126, −156, −157, −167, −169, and −189) elicit similar toxic effects (i.e., immunotoxicity, carcinogenicity, and endocrine disruption) as those caused by tetrachlorodibenzodioxin-2,3,7,8 (TCDD). TCDD is the most potent polychlorinated dibenzodioxin and has significant human toxicity and environmental concerns [22]. These dioxin-like PCB congeners, like TCDD, have a high binding affinity to the aryl hydrocarbon receptor [22]. The World Health Organization (WHO) developed toxic equivalency factors (TEFs) for TCDD-like compounds that can be used to determine the relative potency (toxic equivalents [TEQs]) of dioxin-like compounds for different classes of animals (i.e., fish, birds, and mammals) as well as to facilitate risk assessment for TCDD-like exposure [22,23].

Exposure to PCBs can result in multiple adverse effects in animals. DeLeon et al. 2020 demonstrated that sublethal amounts of PCB exposure resulted in altered sexual behavior in zebra finches, including altered mating song composition and adverse nesting behavior [24]. PCBs have been demonstrated to have significant adverse effects in mammalian wildlife species, including minks, correlation with river otter decline, declining birth rates in bats, and the decreased thickness in harbor seal blubber [25]. According to Montaño et al. 2022, one of the main exposure routes of PCBs for humans is through the ingestion of contaminated food, including consuming contaminated wild game (terrestrial and aquatic species) [14]. Further, a significant exposure route of PCBs can occur through the inhalation of volatized PCBs in contaminated indoor and outdoor air, and atmospheric PCB concentrations are usually higher within industrial settings [15,26,27]. In the current study, our objectives were to document the distribution of PCBs within three feeding guilds (carnivore, herbivore, and omnivore) in and around developed and rural locations in New Mexico. We tested the hypothesis that carnivores and omnivores would contain higher concentrations of the more chlorinated homologs and TEQs than herbivores by examining PCB concentrations in animal biota collected from 2009 to 2025.

## Methods

### Sample collection and third-party chemical analysis

**Fig 1:**
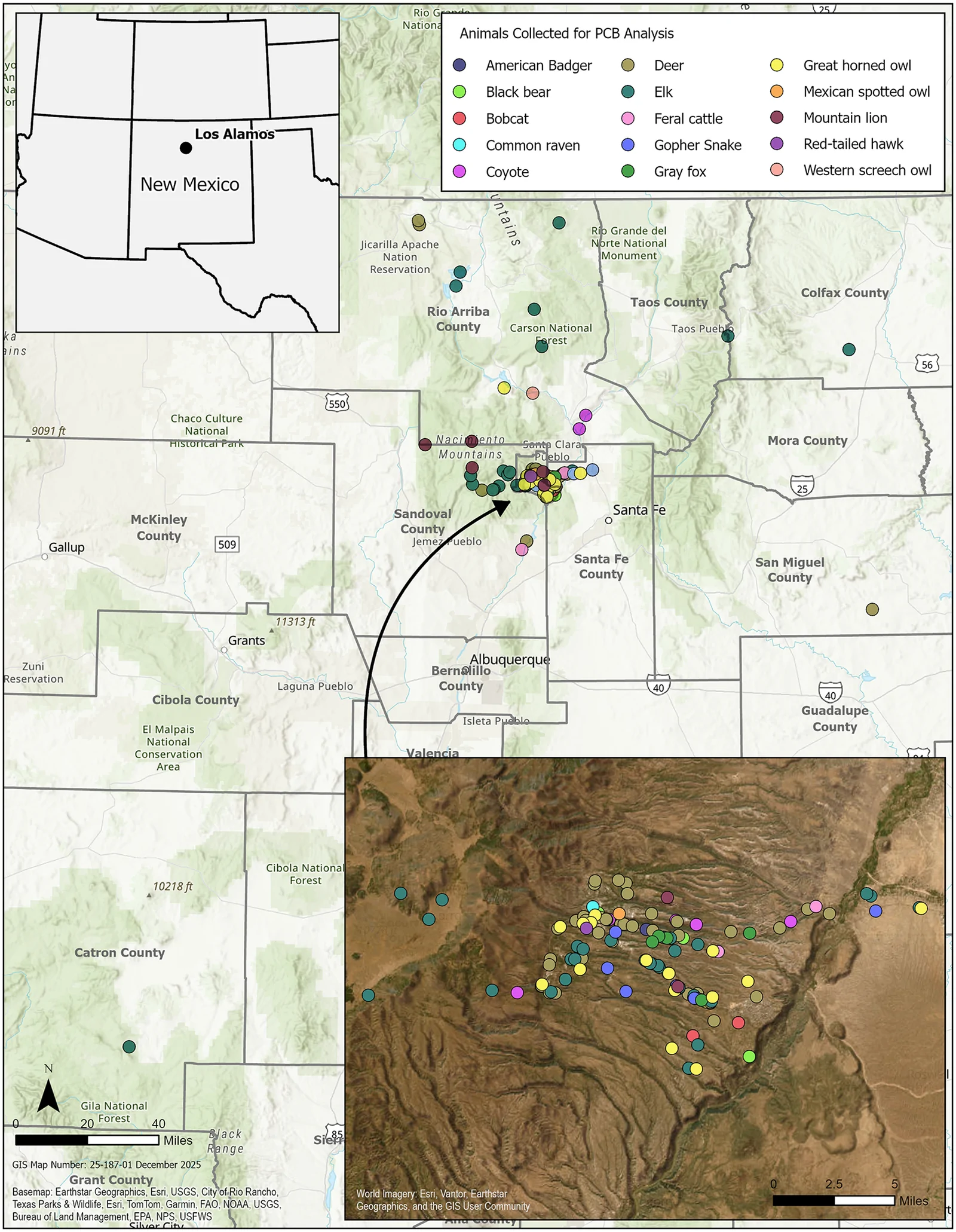
Location of animals opportunistically collected for polychlorinated biphenyls (PCBs) analysis across New Mexico, United States from 2009-2025.

Tissue samples (muscle) were collected opportunistically, with most samples originating from vehicle conflict. Samples include the following: **Mammalian** American badger *(Taxidea taxus*) n = 2, American black bear *(Ursus americanus*) n = 3, bobcat *(Lynx rufus*) n = 2, feral cattle *(Bos taurus*) n = 2, coyote *(Canis latrans*) n = 9, mule deer (deer) *(Odocoileus hemionus*) n = 57, Rocky Mountain elk (elk) *(Cervus canadensis*) n = 43, gray fox *(Urocyon cinereoargenteus*) n = 5, mountain lion *(Puma concolor*) n = 5**, Avian** common raven *(Corvus corax*) n = 1, great horned owl *(Bubo virginianus*) n = 16, Mexican spotted owl (*Strix occidentalis lucida*) n = 1, red-tailed hawk *(Buteo jamaicensis*) n = 1, and western screech owl *(Megascops kennicottii*) n = 1, and **Reptilian** gopher snake *(Pituophis catenifer*) n = 9. Samples were collected as a part of a larger routine and opportunistic monitoring program. All available samples were used within the data analysis. Samples were collected from 2009 to 2025 from multiple locations in New Mexico, United States (Fig 1).

Muscle, rather than fat, was the selected tissue type for PCB analysis in biota due to a human consumption standpoint (i.e., muscle consumption of deer and elk). Samples were collected from developed areas in proximity to Los Alamos and surrounding counties (New Mexico, United States) and from rural (locations that were generally outside of cities, with low population density of less than 2,500 people) locations [28]. When possible, tissue samples were collected at the location where the deceased animal was first located from unexposed (unopened) carcasses. When not possible, the animal or part of the animal was frozen and then processed for the collection of a tissue sample at a later date. Field-dressing tools used in the collection of samples were cleaned between each collection with disinfectants, soap, and water. Mammalian, avian, and reptilian samples were collected under the approved New Mexico Game and Fish Scientific Collection Permits 2864, 3327, and 3865; avian samples were collected under the approved United States Fish and Wildlife Service Permit 24355.

Tissue samples (minimum of 30 g) were placed in individual amber glass jars, sealed with tamper-proof tape, and shipped under full chain of custody to Cape Fear Analytical LLC (Wilmington, North Carolina), which is International Organization for Standardization 17025 accredited and certified analytical laboratory under the Department of Defense Environmental Laboratory Accreditation Program and Department of Energy’s Consolidated Audit Program programs, for PCB analysis by EPA Method 1668. Sample analyses were conducted using methodology outlined in Cape Fear Analytical LLC standard operating procedures. Briefly, Cape Fear Analytical LLC uses EPA approved Method 1668 with laboratory blanks, laboratory control samples, matrix spikes, control sample duplicates for quality control requirements. Prior to operation, multi-level calibration is conducted on the high-resolution mass spectrometer (HRMS). Cape Fear Analytical lab provided data qualifiers for each sample analyzed and each report underwent secondary validation at Los Alamos National Laboratory sample submission office. The secondary validation procedures and data packages for the chain of custody forms can be found on Intellus New Mexico website [29]. Data packages, including internal standard summaries for each sample, can be obtained by visiting www.intellusnm.com, navigating to LANL files within the document folder, and selecting the corresponding year. Sample COC# are listed in the supplemental tables (S1 and S2 Table). PCB concentrations in tissue samples were provided from the Cape Fear Analytical LLC as concentrations of substance per kg of tissue sample. PCB concentrations were downloaded from Intellus New Mexico and all results were reviewed by staff (S1 Table), including validation qualifiers and validation codes.

### Polychlorinated biphenyls analysis based on feeding guild

Tissue samples were grouped based on feeding guilds: **herbivores** (deer, elk, and cattle), **omnivores** (bear, coyote, gray fox, and common raven), and **carnivores** (American badger, bobcat, gopher snake, great horned owl, Mexican spotted owl, mountain lion, red-tailed hawk, and western screech owl). The total number of samples used in this part of the investigation was 158 (37 carnivores, 103 herbivores, and 18 omnivores). Data were compiled from the results of PCBs found in animal tissue samples collected from 2009 through 2025. Within the dataset, seven samples were submitted as field duplicates to assess the accuracy of analysis. Results from these duplicate samples were averaged into one sample from each animal. Main and interaction effects were determined using Analysis of Variance (ANOVA) with a nonparametric Wilcoxon comparison (α = 0.05); data analysis was performed in JMP [30]. An ANOVA with nonparametric Wilcoxon comparison was conducted to determine if the concentration of PCB homologs and total PCB concentrations differed among feeding guilds. Analysis of Variance was run only on sample sets with greater than 20 percent positive detection rate for the PCB homolog analyses using Wilcoxon comparison [31]. Data were transformed log (concentration+1) to help with data visualization. Percentages of PCB homologs and dioxin-like PCBs by feeding guilds were plotted using GraphPad Prism 10.4.1. [32].

Additionally, two separate non-metric multidimensional scaling (NMDS) analyses with Bray-Curtis dissimilarity matrix were run to assess overall differences in PCB homologs and dioxin-like PCBs from animals in relation to the three different feeding guilds—herbivores, omnivores, and carnivores. For both analyses, the appropriate number of dimensions was determined by plotting final stress versus the number of dimensions and choosing the number of axes beyond which reductions in stress were small. The NMDS results were tested for differences in PCB homologs and dioxin-like PCBs for feeding guilds with a nonparametric pairwise comparison statistical method (PERMANOVA). The variables that were driving the distribution patterns (p < 0.05) relative to feeding guilds were investigated using the R envfit function in the R vegan package v.2.6.8, which uses a permutation test to evaluate significance, R statistical software version 4.4.1 [33,34].

Toxic equivalents were calculated by multiplying the concentration of each of the 12 individual dioxin-like PCBs (PCB −77, −81, −105, −114, −118, −123, −126, −156, −157, −167, −169, and −189 (S2 Table)) with their respective toxic equivalency factors (TEFs), then summed for all individual samples. Mammal TEFs were compiled from Van den Berg et al. 2006, and bird TEFs were compiled from Van den Berg et al. 1998 [22,23]. No TEFs are available for reptiles; however, fish TEFs from Van den Berg et al. 1998 were used as a surrogate (as both fish and reptiles are ectotherms) [23]. PCB-156 and PCB-157 co-eluted and therefore are reported as a total of the two congeners; however, the TEFs for these two congeners were the same across taxa. Due to the high rate of non-detects, both a TEQ minimum and a TEQ maximum were calculated for each individual sample, where non-detects were set to 0 for the TEQ minimum calculation and non-detects were set at the limit of quantitation for the TEQ maximum calculation [35]. TEQ minimum and TEQ maximum datasets were assessed for normality with the Shapiro-Wilk test. Then, a Kruskal-Wallis test with a Dunn’s post-hoc pairwise comparison was run to assess differences in TEQ minimum and TEQ maximum among feeding guilds [32]. One deer sample was omitted because the dioxin-like PCB congeners were reported inconsistently from all of the other samples (i.e., PCB-118 was not reported and PCB-156 and PCB-157 were reported individually, rather than co-eluting).

### Magnification of polychlorinated biphenyls within the assessed New Mexico food web

To aid in the visualization of potential PCB transfer across trophic levels, a food web was constructed from the collected samples (n ≤ 3 of a species to be included; human, small mammals, and vegetation images were used only as visual representation) in Visio 365 software [36]. A magnification factor was quantified using a linear regression of log_10_-transformed PCB concentrations against assigned trophic levels, with a magnification factor > 1 indicating biomagnification across trophic levels. For this analysis, stable isotope data were unavailable, so we used trophic position estimates from ecological studies as a substitute [37,38]: 2.0 for herbivores, 2.8 for omnivores, and 3.8 for carnivores. Trophic levels were treated as a continuous variable [37,38]. The ratio of PCB concentrations among the carnivore, omnivore, and herbivore feeding guilds was also calculated to further examine the magnification of PCBs.

In addition, biomagnification factors (BMFs) were calculated on a subset of data to evaluate trophic transfer of PCB homolog groups from ungulate prey (elk and mule deer) to a top predator (mountain lion). PCB concentrations (µg/kg ww) were averaged within each species group for all quantified homolog classes (monochloro through decachloro). For each homolog class, BMF values were computed as the ratio of the mountain lion (n = 5) mean concentration to the mean concentration of combined elk (n = 43) and deer (n = 57) prey. Due to the small sample sizes of mountain lions and to estimate uncertainty around these ratios, nonparametric bootstrapping was performed by resampling individuals within predator and prey groups with replacement for 5,000 iterations. For each iteration, homolog-specific BMF values were recalculated. Median BMFs and 95 percent bootstrap confidence intervals (CIs; 2.5th–97.5th percentiles) were generated for each homolog class. Biomagnification was inferred when BMF values exceeded 1 and when the associated CI did not overlap the threshold of 1.

### Polychlorinated biphenyls analysis based on location

Tissue samples were separated based on sample collection sites and broken into two categories: developed and rural locations. To run a balanced analysis, the difference in total PCB concentrations within deer and elk muscle were examined. Deer and elk were selected for this analysis because they were the species that had the largest sample sizes. Main and interaction effects for deer and elk samples were determined using an ANOVA, with a nonparametric Wilcoxon comparison (α = 0.05) between locations to determine if the total PCB concentrations in deer and elk muscle were different between locations [28]. Data were transformed log (concentration+1) to help with the visualization of the data. Trends over time were plotted for deer and elk from developed and rural areas using JMP [30]. A complementary NMDS subset analysis was conducted on elk samples to assess overall differences in PCB homolog concentrations between developed and rural locations to further validate the findings [33]. NMDS results for differences in PCB homolog concentrations with a nonparametric pairwise comparison statistical method (PERMANOVA) were tested. The variables that were driving the distribution patterns (P < 0.05) relative to feeding guilds were investigated using the R envfit function in the R vegan package v.2.6.8 [33], which uses a permutation test to evaluate significance [33].

## Results

### Polychlorinated biphenyls analysis based on feeding guild

**Fig 2:**
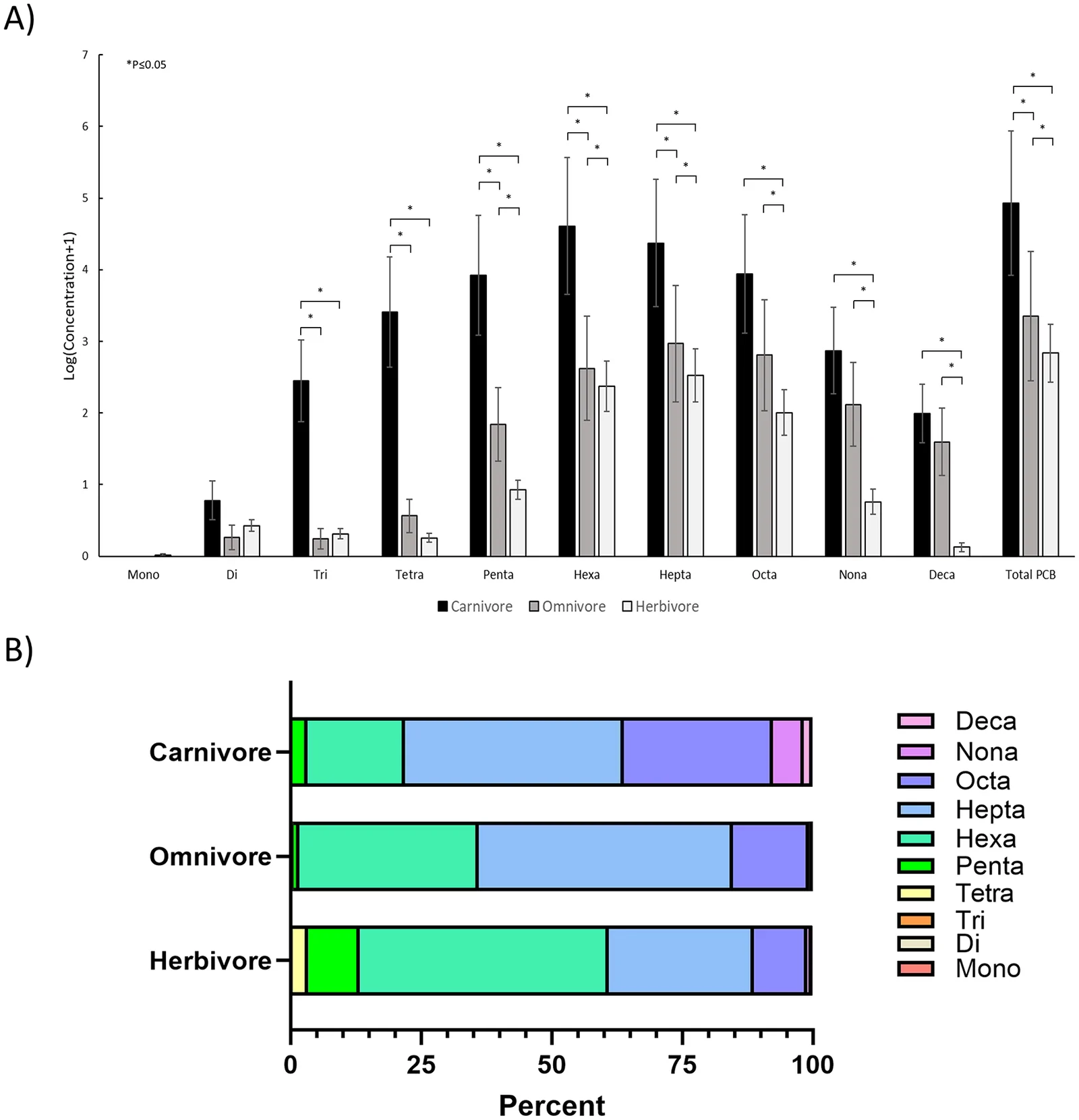
Polychlorinated biphenyls (PCB) homolog concentrations. A) Log transformed average concentrations (ng/kg tissue + 1) of polychlorinated biphenyls (PCBs) homolog concentrations + SE within different feeding guilds. *ng/kg was used instead of µg/kg for ease of visualization. B) Percentage of PCB homologs by feeding guild.

The concentration of total PCB homologs varied significantly (P < 0.05) among feeding guilds. When tissue samples were grouped based on feeding guild (carnivore, omnivore, and herbivore), carnivores (mean ± STD 85.16 ± 278.12, median 9.24, 75 percent quantiles 42.7, 25 percent quantiles 0.78 µg/kg) had significantly more total PCB concentrations than either omnivores (mean ± STD 2.23 ± 3.18, median 0.713, 75 percent quantiles 2.87, 25 percent quantiles 0.33 µg/kg) or herbivores (mean ± STD 0.68 ± 6.23, median 0.019, 75 percent quantiles 0.054, 25 percent quantiles 0.002 µg/kg) feeding guilds (carnivore vs. omnivore [P = 0.0042] and carnivore vs. herbivore [P < 0.0001]). The omnivore feeding guild also had higher concentrations of total PCBs when compared with the herbivore feeding guild (P < 0.0001). When PCB concentrations for individual homologs were examined based on feeding guilds, the carnivore feeding guild had higher concentrations of trichloro, tetrachloro, pentachloro, hexachloro, heptachloro, octachloro, nanochloro, and decachloro homologs compared with the herbivore feeding guild and higher levels of trichloro, tetrachloro, pentachloro, hexachloro, and heptachloro homologs compared with the omnivore feeding guild. The omnivore feeding guild had higher concentrations of pentachloro, hexachloro, heptachloro, octachloro, nanochloro, and decachloro homologs compared with the herbivore feeding guild. No significant elevated differences of PCB homologs were found in the herbivore feeding guild compared with either the carnivore or omnivore guilds (Fig 2, Table 1).

**Table 1:** A) Average concentrations (µg/kg tissue) of polychlorinated biphenyls (PCBs) homologs (chlorobiphenyl) among different feeding guilds. B) Statistical analysis (ANOVA with a nonparametric Wilcoxon comparison) examining differences among feeding guilds. Bolded values represent significantly different values (P < 0.05).

**A)**				
**PCB Homolog**	**Carnivore (µg/kg)**	**Omnivore (µg/kg)**	**Herbivore (µg/kg)**	
Monochloro	0	0	0.00004	
Dichloro	0.005	0	0.0016	
Trichloro	0.2809	0.0007	0.001	
Tetrachloro	2.58	0.0026	0.0008	
Pentachloro	8.396	0.0681	0.0074	
Hexachloro	40.59	0.417	0.235	
Heptachloro	23.63	0.935	0.333	
Octachloro	8.763	0.638	0.1	
Nonachloro	0.7427	0.131	0.0047	
Decachloro	0.097	0.0385	0.0003	
Total PCBs	85.16	2.234	0.684	
**B)**				
**PCB Homolog**	**Carnivore > Herbivore**	**Carnivore > Omnivore**	**Omnivore > Herbivore**	**Omnivore < Herbivore**
Trichloro	**0.0002**	**0.0138**	NA	0.6337
Tetrachloro	**<.0001**	**0.0005**	0.0783	NA
Pentachloro	**<.0001**	**0.0001**	**<.0001**	NA
Hexachloro	**<.0001**	**0.0001**	**<.0001**	NA
Heptachloro	**<.0001**	**0.0143**	**<.0001**	NA
Octachloro	**<.0001**	0.1144	**<.0001**	NA
Nonachloro	**<.0001**	0.3282	**<.0001**	NA
Decachloro	**<.0001**	0.5712	**<.0001**	NA
Total PCBs	**<.0001**	**0.0042**	**<.0001**	NA

**Fig 3:**
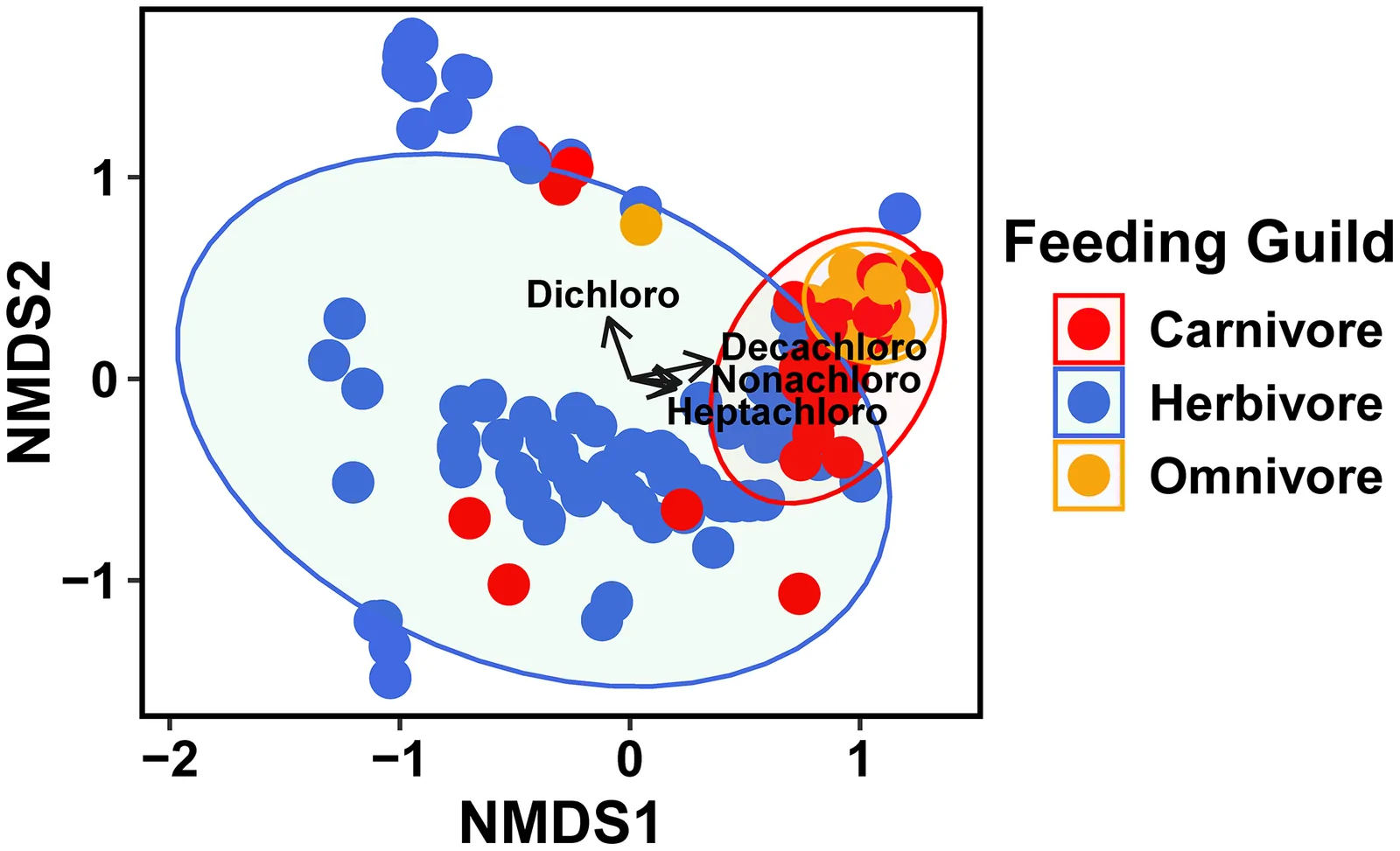
Nonmetric multidimensional scaling (NMDS) of polychlorinated biphenyls (PCBs) homolog composition in three different feeding guilds. Pairwise PERMANOVA (adonis) indicated significant differences in PCB composition among dietary groups; carnivores differed from herbivores (R^2^ = 0.10, F = 16.22, P = 0.003) and omnivores (R^2^ = 0.08, F = 4.72, P = 0.009), and herbivores also differed from omnivores (R^2^ = 0.08, F = 9.61, P = 0.003). Vector arrows show significant variables (PCBs) and the direction of significant influence driving the distribution patterns in the NMDS (P < 0.05).

Nonmetric multidimensional scaling results showed differences in the three feeding guilds in relation to PCB homologs (Fig 3). To visualize the PCB homolog associations with different feeding guilds, PCB homologs were overlaid based on weighted average abundance across sites onto the NMDS biplot. Pairwise PERMANOVA revealed significant differences among all feeding guilds, with carnivores differing from herbivores (R^2^ = 0.10, F = 16.22, P = 0.003) and omnivores (R^2^ = 0.08, F = 4.72, P = 0.009), as well as a significant difference between herbivores and omnivores (R^2^ = 0.08, F = 9.61, P = 0.003). The differences calculated in the NMDS and seen in the herbivore PCB homologs compared with the carnivore and omnivore PCB homologs were significantly driven by dichloro, decachloro, nonachloro, and heptachloro homolog detections (P < 0.05; Fig 3).

**Fig 4:**
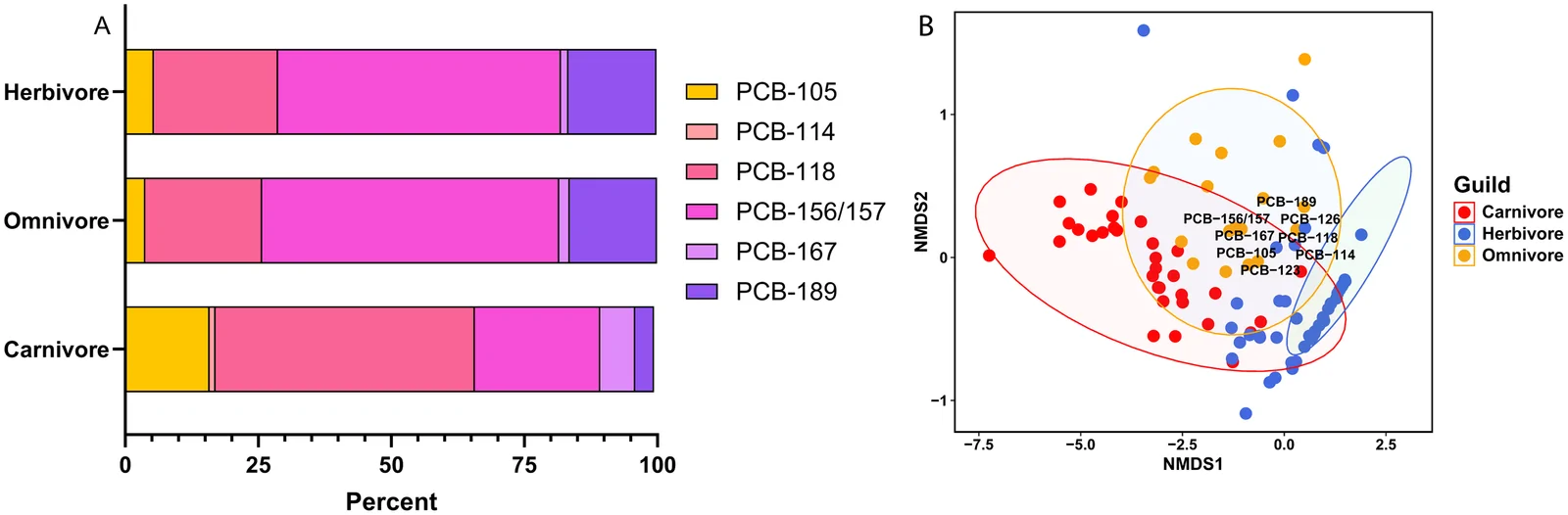
A) Percentage of dioxin-like polychlorinated biphenyls (PCB) congeners by concentrations (only congeners with concentrations greater than 1 percent of the total sum are displayed by feeding guild). B) Nonmetric multidimensional scaling (NMDS) of select dioxin-like PCB congeners in three different feeding guilds. Pairwise PERMANOVA (adonis) indicated significant differences in PCB composition among feeding guilds. Pairwise PERMANOVA (adonis) indicated significant differences between carnivores and herbivores (R^2^ = 0.25, F = 44.65, P = 0.003) and omnivores (R^2^ = 0.12, F = 7.16, P = 0.003), and herbivores also differed from omnivores (R^2^ = 0.21, F = 31.30, P = 0.003). Note, PCB-156 and PCB-157 co-eluted and are therefore reported as PCB-156/157.

All dioxin-like PCB congeners were detected in at least one tissue sample; however, PCB-77, PCB-81, PCB-123, PCB-126, and PCB-169 were detected at concentrations less than 1 percent of the total sum. The composition of dioxin-like PCB congeners among feeding guilds was similar between herbivores and omnivores, with the most dominant congers belonging to PCB-156/157 (the “/” between PCB-156 and PCB-157 indicates co-elution within the sample), PCB-118, and PCB-189 respectively. In carnivores, the most dominant congeners were PCB-118, PCB-156/157, and PCB-105 (Fig 4A). The NMDS subset analysis for dioxin-like PCBs also showed differences among the three feeding guilds where pairwise PERMANOVA (adonis) indicated significant differences in carnivores from herbivores (R^2^ = 0.25, F = 44.65, P = 0.003) and omnivores (R^2^ = 0.12, F = 7.16, P = 0.003), and herbivores also differed from omnivores (R^2^ = 0.21, F = 31.30, P = 0.003). The differences seen in the herbivore dioxin-like PCB concentrations compared with the carnivores and omnivores were driven by PCB-105, PCB-114, PCB-118, PCB-123, PCB-126, PCB-156/157, PCB-167, and PCB-189, detections (P < 0.05; Fig 4B).

Both TEQ minimum and TEQ maximum calculations revealed similar observations, where herbivores contained the lowest TEQs concentrations and carnivores contained the highest TEQs concentrations (herbivore < omnivore < carnivore; Table 2). The Kruskal-Wallis tests revealed significant differences among feeding guilds for TEQ minimum (P < 0.0001) and TEQ maximum (P < 0.01). Dunn’s post-hoc pairwise comparison tests revealed that the TEQ minimum differed between herbivores and omnivores (P < 0.0001) and herbivores and carnivores (P < 0.0001); the TEQ maximum differed only between herbivores and carnivores (P < 0.01; Table 2).

**Table 2:** Median and interquartile range of toxic equivalent (TEQ) concentrations (pg/g) among feeding guilds.

Feeding Guild	N	TEQ minimum (pg/g)			TEQ maximum (pg/g)		
		median^1^	25%	75%	median	25%	75%
Herbivore	102	0.0000^B^	0.0000	0.0001	0.1888^B^	0.0849	0.1982
Omnivore	18	0.0023^A^	0.0007	0.0086	0.1998^AB^	0.0884	0.5377
Carnivore	37	0.0843^A^	0.0035	0.6686	0.5557^A^	0.0869	3.573

^1^Median values with different uppercase letter superscripts among feeding guilds differ significantly by Dunn’s post-hoc pairwise comparison tests.

### Magnification of polychlorinated biphenyls within the assessed New Mexico food web

**Fig 5:**
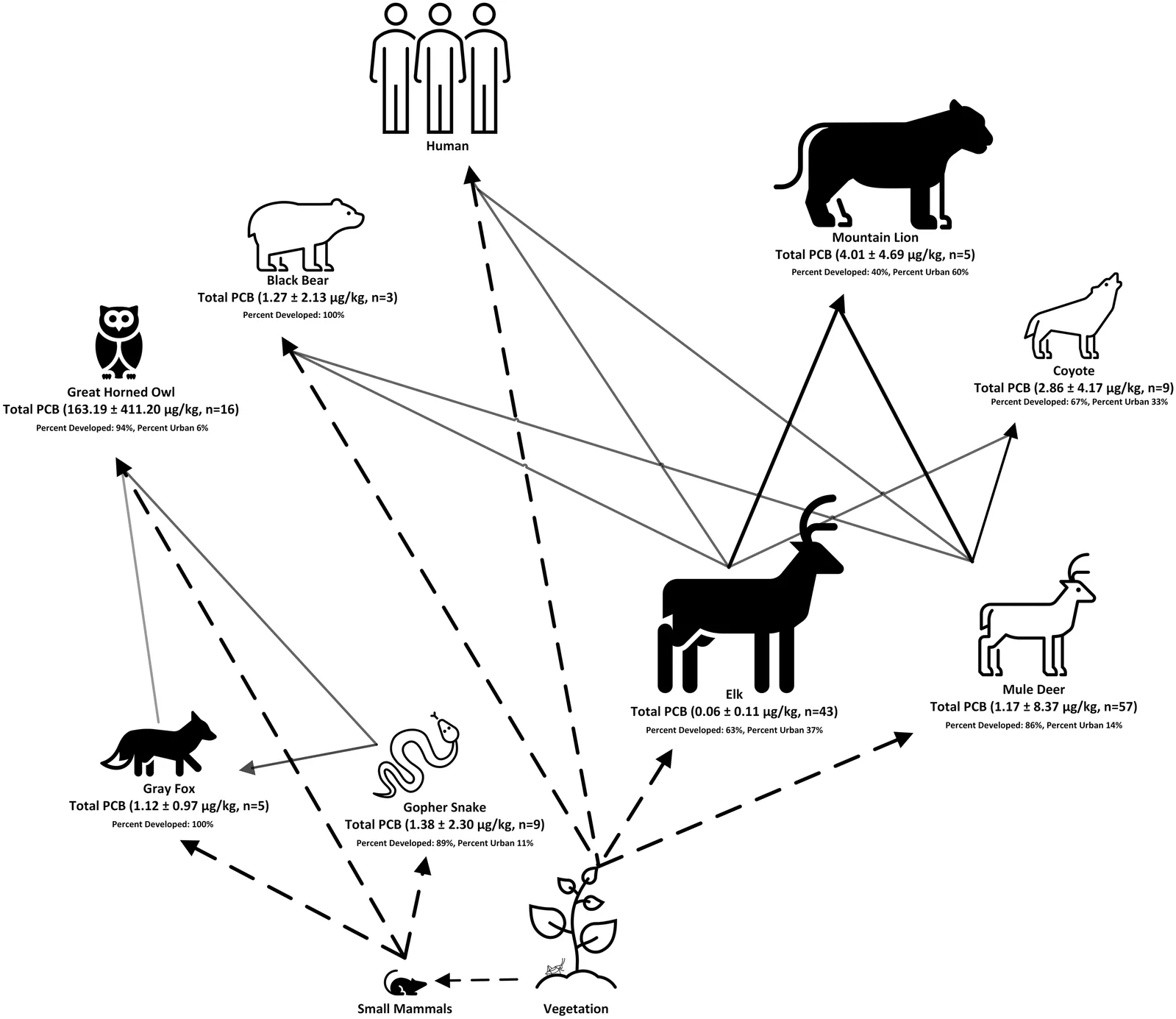
Polychlorinated biphenyls (PCBs) concentrations within the evaluated food web in New Mexico, United States. Food web is based on primary consumption pathways.

From the 158 samples collected, 9 species (coyote, black bear, elk, gray fox, great horned owl, gopher snake, mountain lion, and deer) had three or more replicates. From these species, a food web that depicts the movement of PCBs in the environment was composed (Fig 5).

**Fig 6:**
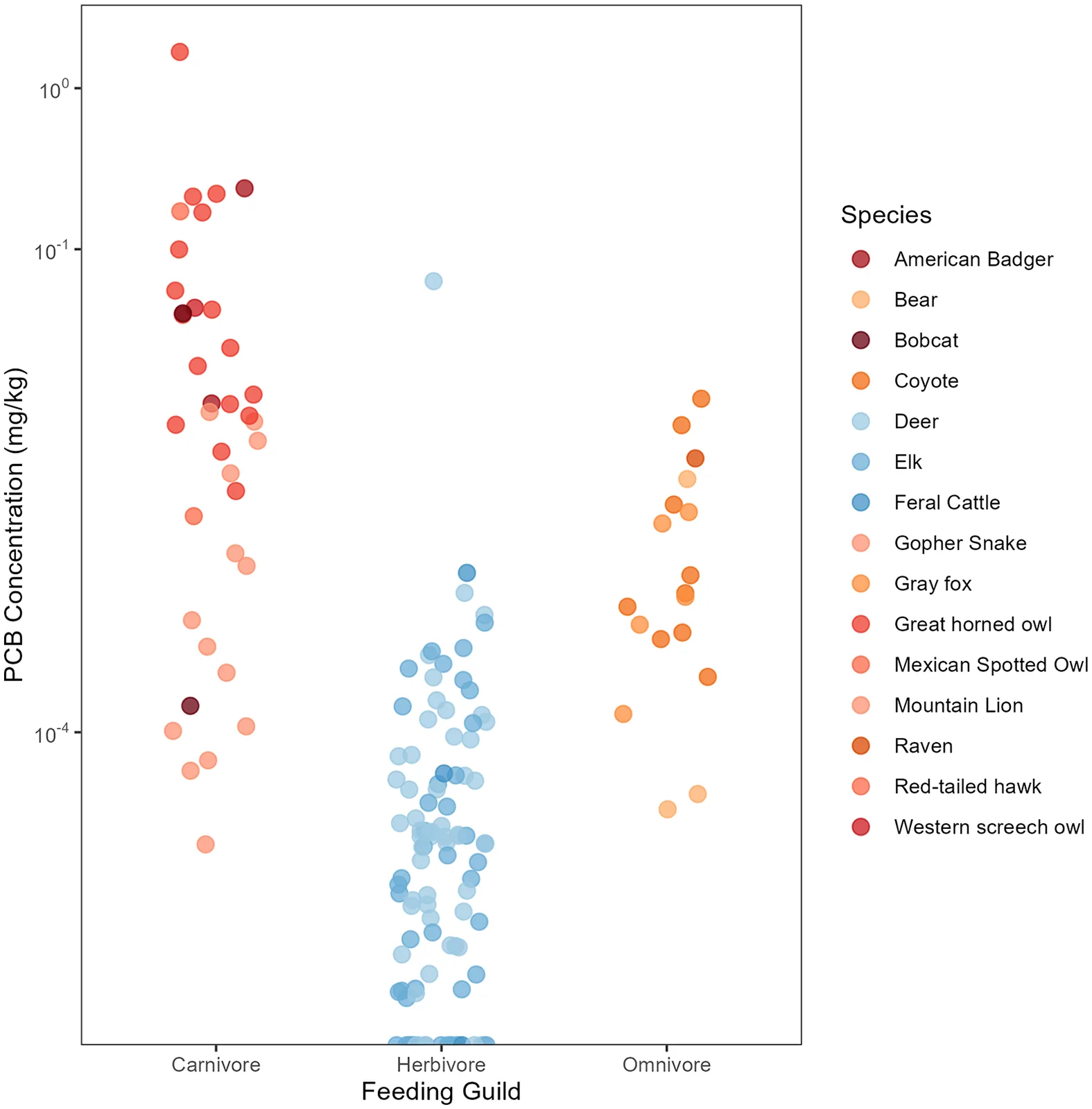
Total polychlorinated biphenyls (PCBs) mg/kg concentrations grouped by different feeding guilds.

To evaluate PCB magnification among feeding guilds, total PCB concentrations were regressed against assigned trophic position (2.0 for herbivores, 2.8 for omnivores, and 3.8 for carnivores) [37,38]. The magnification factor resulted in a value of 30.09, which indicated that PCB concentrations increased significantly with trophic level position, providing strong evidence that PCB concentrations increased across successive feeding guilds [39,40]. To expand on the magnification among trophic levels, ratios among each feeding guild were generated to examine the overall increase in PCB concentrations among feeding guilds. Overall, omnivores had 3.27 times higher concentrations of total PCBs than herbivores, and carnivores had 124.5 times higher concentrations of total PCBs than herbivores and 38.1 times higher concentrations of total PCBs than omnivores (Fig 6).

We further chose to examine BMF between predator (mountain lions) and prey (elk and deer) samples (based on sample sizes and verified dietary pathways). Mountain lions exhibited clear biomagnification of mid- to high-chlorinated PCB homologs relative to their ungulate prey. Strong biomagnification was observed for pentachloro, nonachloro, and decachloro (BMFs ranging from 43.5 to 278.4), with decachloro showing the greatest accumulation (Table 3).

**Table 3:** Biomagnification factors (BMFs) for polychlorinated biphenyls (PCBs) homolog groups from ungulate prey (elk and deer) to mountain lions. BMFs were calculated as the ratio of mean wet-weight (ww) PCB concentrations (µg/kg ww) in mountain lions (n = 5) to the combined mean concentrations in elk (n = 43) and deer (n = 57) for each PCB homolog.

Predator	Prey	Homolog Category	BMF µg/kg of Predator/µg/kg of Prey	Biomagnification? BMF > 1 = Yes BMF < 1 = No
Mountain Lion	Elk + Deer	Monochloro	0	No
Mountain Lion	Elk + Deer	Dichloro	0	No
Mountain Lion	Elk + Deer	Trichloro	0	No
Mountain Lion	Elk + Deer	Tetrachloro	**1.975**	Yes
Mountain Lion	Elk + Deer	Pentachloro	**43.51**	Yes
Mountain Lion	Elk + Deer	Hexachloro	**6.756**	Yes
Mountain Lion	Elk + Deer	Heptachloro	**5.337**	Yes
Mountain Lion	Elk + Deer	Octachloro	**8.801**	Yes
Mountain Lion	Elk + Deer	Nonachloro	**49.42**	Yes
Mountain Lion	Elk + Deer	Decachloro	**278.4**	Yes

**Fig 7:**
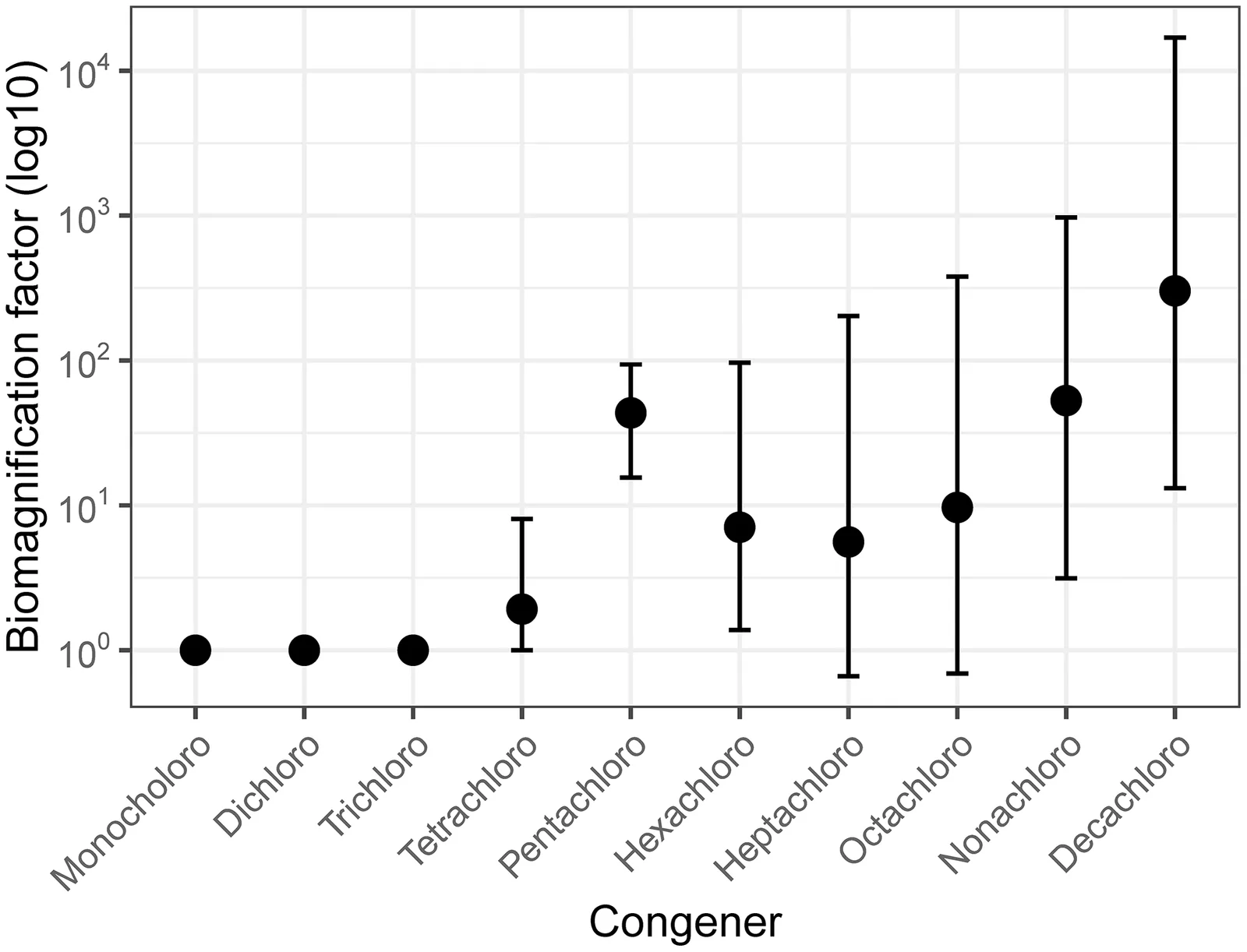
Median biomagnification thresholds (BMFs) and 95 percent bootstrap confidence intervals (CIs; 2.5th–97.5th percentiles) were generated for each polychlorinated biphenyls (PCBs) homolog between mountain lion from elk and deer.

Confidence intervals for penta, hexa, nona, and deca homologs consistently remained above the biomagnification threshold (BMF = 1), confirming robust trophic magnification (Fig 7). In contrast, lower-chlorinated homologs (mono, di, and tri) showed no biomagnification (BMF < 1), consistent with their higher metabolic turnover and reduced persistence in tissues (Fig 7).

### Polychlorinated biphenyls analysis based on location

The total concentration of PCBs in the muscle of elk and deer were significantly different between rural and developed locations (P < 0.0001 and P = 0.0206, respectively). When we examined the total concentration of PCBs found within muscle tissue in the herbivore (only deer or elk) feeding guild, we noted that the average concentrations found within tissue collected from rural deer samples was 0.011 ± 0.018 µg/kg tissue (median 0.002, 75 percent quantiles 0.02, 25 percent quantiles 0 µg/kg) and developed deer samples was 1.36 ± 9.03 µg/kg tissue (median 0.0227, 75 percent quantiles 0.0812, 25 percent quantiles 0.007 µg/kg; Table 4), which were significantly different (P = 0.0206) from each other. In addition, tissue collected from rural elk samples was 0.0035 ± 0.0068 µg/kg tissue (median 0.000, 75 percent quantiles 0.004, 25 percent quantiles 0 µg/kg) and developed elk samples 0.097 ± 0.131 µg/kg tissue (median 0.022, 75 percent quantiles 0.182, 25percent quantiles 0.006 µg/kg; Table 4), which were significantly different (P < 0.0001) from each other.

**Table 4:** Average, standard deviation, and median concentrations of polychlorinated biphenyls (PCBs) in deer and elk from rural and developed locations in New Mexico.

	Rural				Developed			
Species	Avg (µg/kg)	STDEV (µg/kg)	Median (µg/kg)	N	Avg (µg/kg)	STDEV (µg/kg)	Median (µg/kg)	N
Deer	0.011	0.0181	0.0109	8	1.36	9.034	0.0227	49
Elk	0.0035	0.0068	0.0000	16	0.097	0.131	0.0228	27

*Note: Food and Drug Administration (FDA) advisory levels of 3,000 µg/kg fat basis.

**Fig 8:**
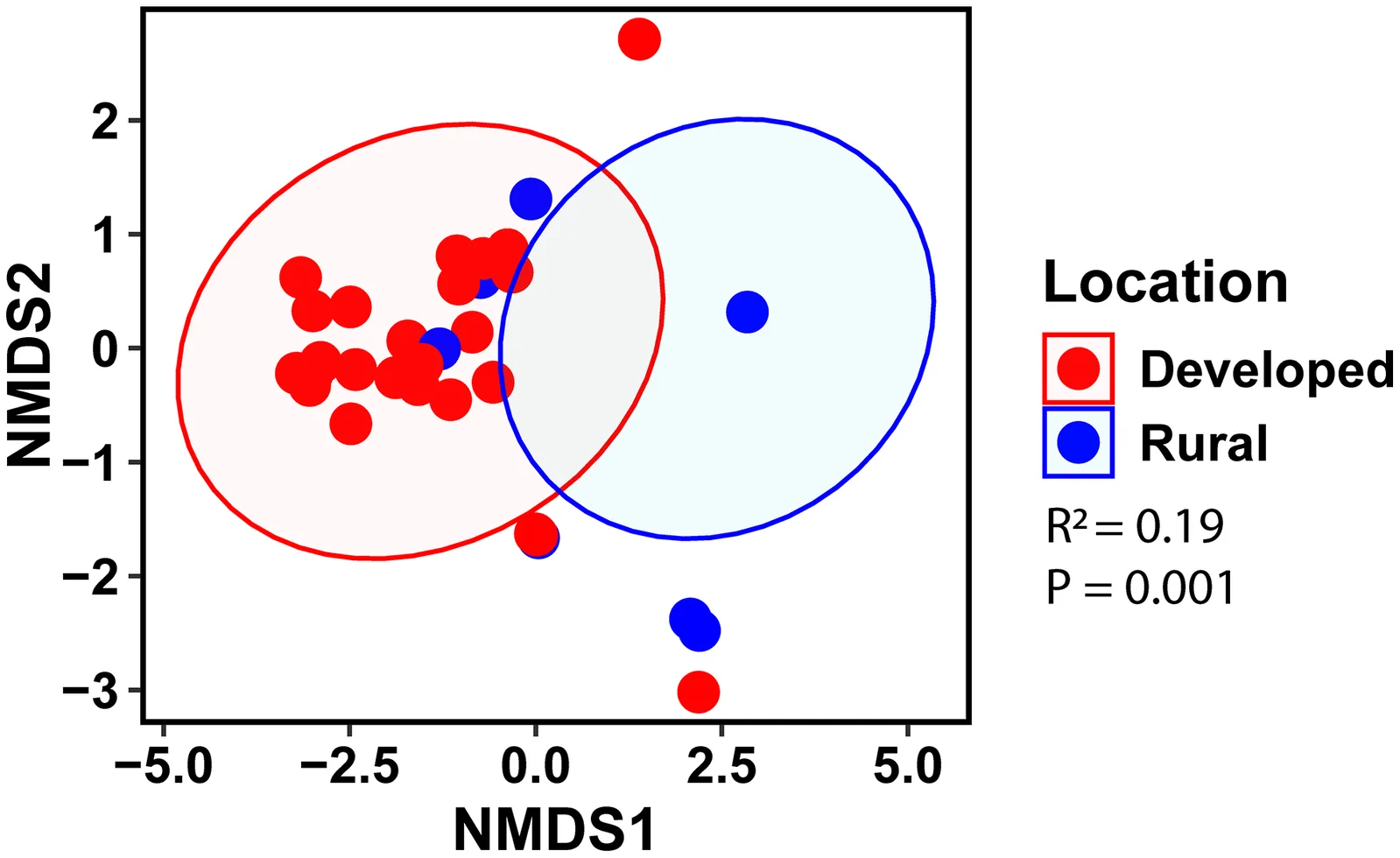
Nonmetric multidimensional scaling (NMDS) of PCB homolog compositions in elk samples collected from developed and rural locations (PERMANOVA, F = 9.31, R^2^ = 0.19, P = 0.001).

An NMDS analysis further demonstrated differences between elk sampled from developed and rural locations in relation to PCB homolog concentrations (PERMANOVA, F = 9.31, R^2^ = 0.19, P = 0.001; Fig 8).

**Fig 9:**
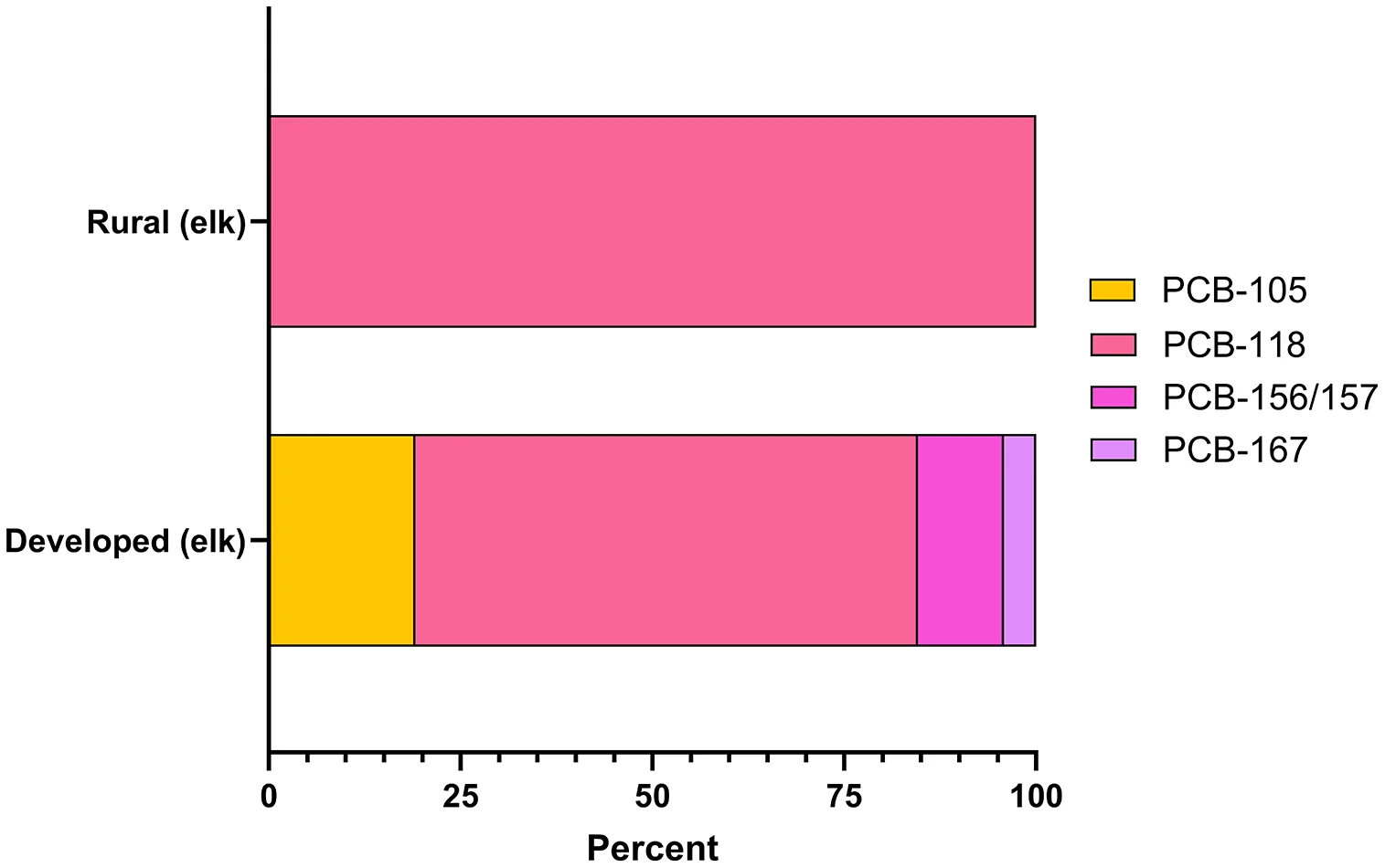
Percentage of dioxin-like PCB congeners by concentrations (only congeners with concentrations greater than 1 percent of the total sum are displayed) for elk collected from rural and developed locations. Note: PCB-156 and PCB-157 co-eluted and are therefore reported as PCB-156/157.

Dioxin-like PCB composition in elk differed from rural and developed areas. Elk sampled from rural locations only contained PCB-118; PCB-118 was the dominant congener also detected in elk from developed areas. Greater diversity existed in dioxin-like PCB congeners detected in developed locations, including PCB-105, PCB-156/157, and PCB-167 (Fig 9).

**Fig 10:**
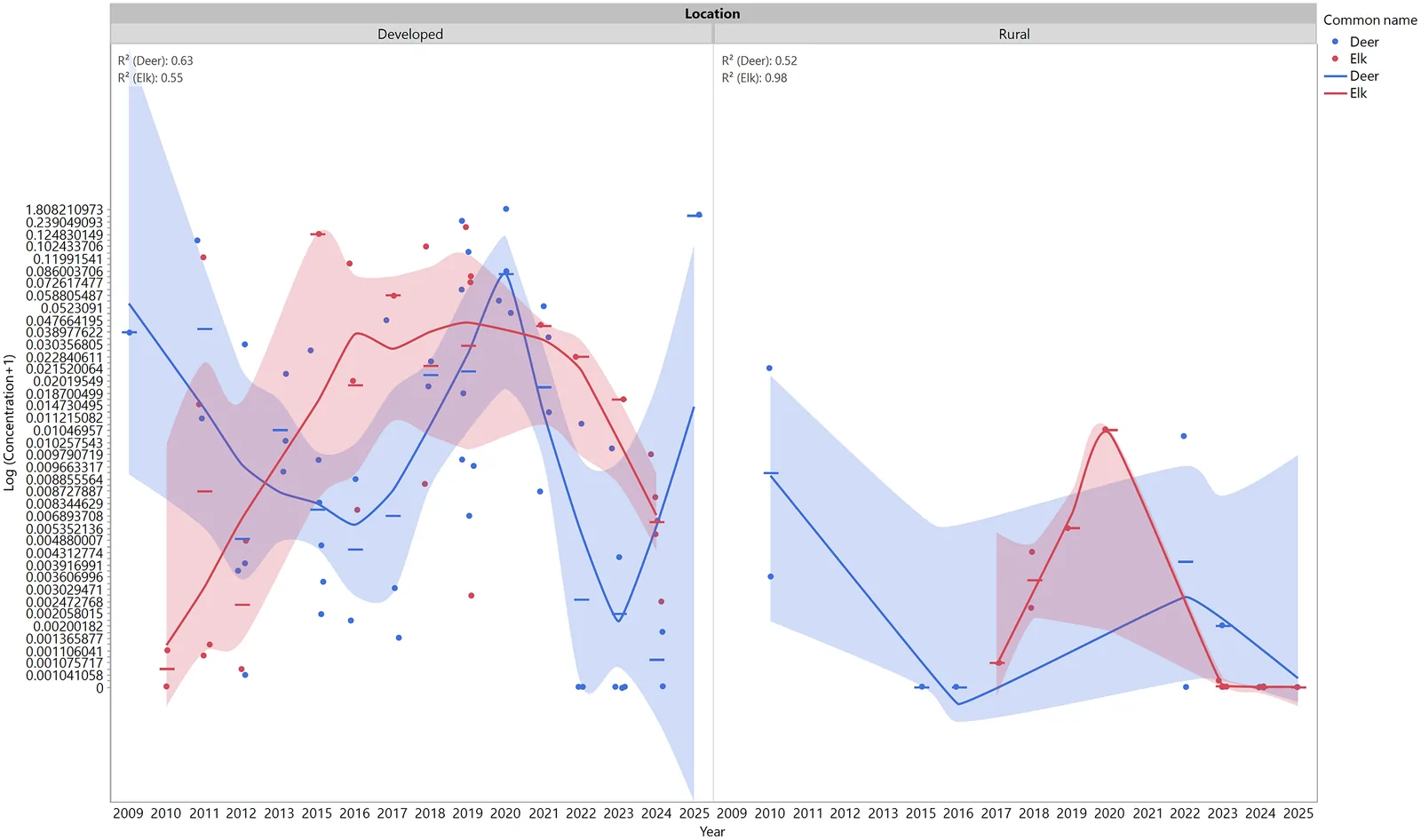
Elk and deer trend analyses from 2009 through 2025. Data represents the log-transformed mean concentrations (µg/kg tissue + 1) of total PCBs within elk and deer muscle.

When log-transformed mean concentrations of total PCBs were graphed between sampling areas, we noted that no specific pattern of increasing or decreasing concentrations of PCBs occurred over time (Fig 10). The data also demonstrated notable variations with concentrations between samples collected in both developed and rural locations.

Within the developed location, one sample (a deer collected in Los Alamos township) had elevated levels of PCBs (63.3 µg/kg), which was two orders of magnitude higher than the next highest detection. (This detection was left in the analysis as it was reported as a true detection by Cape Fear Analytical). This elevated detection led to larger-than-anticipated standard deviation in the developed sampling location. Although the average rural and developed values for deer were higher compared with the elk values, there was no statistical significance between animal species (P = 0.5723 developed, P = 0.4317 rural).

## Discussion

Within this investigation, we examined the distribution of PCB concentrations among biota found in New Mexico, which we separated into three feeding guilds (carnivore, omnivore, and herbivore) and two locations (rural and developed). We hypothesized that we would see more total accumulation of PCBs, chlorinated PCBs, and TEQs in higher trophic levels (carnivores). When we examined trophic levels of animals found within the environment in New Mexico, we noted that the carnivore feeding guild had significantly more total PCBs within their tissue than either of the omnivore or the herbivore feeding guilds. We also noted that the higher trophic-level organisms (carnivores) overall possessed more of each PCB homolog and higher concentrations of TEQs. This result included the biomagnification of total PCBs up trophic levels, resulting in omnivores having 3.27 times higher concentrations of total PCBs than herbivores; carnivores having 124.5 times higher concentrations of total PCBs than herbivores and 38.1 times higher concentrations of total PCBs than omnivores. Our NMDS ordination and PERMANOVA analyses demonstrated clear separation among feeding guilds, indicating that PCB homolog profiles differ significantly with diet. In particular, carnivores and omnivores were distinguished from herbivores by greater contributions of hepta-, nona-, and decachloro PCB homologs, suggesting feeding guild–specific patterns of PCB exposure or accumulation. Total PCB concentrations and the proportions of dioxin-like PCBs seemed to be influenced by location, with higher total PCB concentrations and dioxin-like PCBs being found in developed areas. The findings of this investigation provide significant insight into PCB concentrations collected in developed areas when compared with rural locations and the distribution, composition, and biomagnification of PCBs in terrestrial animals in New Mexico.

Within each feeding guild, there was a bell-shaped distribution of PCB homologs, with the highest average abundance of homologs being partially chlorinated, suggesting that the most prevalent PCB homologs within tissue samples are not the most chlorinated compounds but instead are the partially chlorinated compounds. This result is most likely due to the overall use of different chlorinated compounds, with the highly chlorinated compounds being used less frequently than the partially chlorinated compounds in industrial processes. For example, Aroclor 1260 (the PCB mix that was commonly used in transformers and hydraulic fluids) has an average chlorine content of 60 percent, with the highest homologs belonging to pentachloro (43.35 percent) and hexachloro (38.54 percent) [41]; however, within our dataset, the tetra, penta, and hexa congeners (which contain 10 of the 12 dioxin-like highly toxic congeners) had some of the highest abundances in the carnivore feeding guild [42]. Studies of dioxin-like PCBs in terrestrial animals are minimal; however, our observations in the proportions of dioxin-like PCBs in muscle samples are similar to those observed in muscle samples of three different species of wild game reported in Warenki-Bany et al. 2016, where congeners PCB-105, PCB-118, PCB-156, PCB-167, and PCB-189 were observed at higher concentrations relative to other dioxin-like PCB congeners [43]. In this study, the dominant dioxin-like PCB congeners were PCB-118, PCB-156/157 and PCB-189 in herbivores and omnivores and PCB-105, PCB-118, and PCB-156/157 in carnivores.

PCB accumulation is a major concern in aquatic ecosystems, especially in higher-trophic-level organisms; however, limited work examining PCB concentrations in terrestrial biota has been conducted outside of Europe [44]. When examining PCB concentrations within terrestrial wildlife collected from multiple regions in Poland, Warenik-Bany noted the PCB concentrations in the muscle of red deer at 6.21 ± 3.71 µg/kg fat and 7.70 ± 6.92 µg/kg fat [43]. We converted their results to a wet basis (concentration in fat = concentration wet/percent fat, [fat percentage noted 1–11.5 percent in deer muscle within their investigation]) resulting in red deer concentrations of 0.06–0.714 µg/kg ww and roe deer concentrations of 0.077–0.885 µg/kg ww [43]. This conversion allowed for a comparison with the wet weight PCB concentrations reported in this study. The average of total PCBs collected within our investigation for deer (1.17 µg/kg ww) and elk (0. 06 µg/kg ww) was similar to what Warenik-Bany found in their investigation [43]. We also compared our findings to the United States Food and Drug Administration (FDA) standard of 3,000 µg/kg fat basis for red meat consumption by humans, direct comparisons are challenging because FDA regulations are on a fat basis in red meat and our samples are based on wet weight (PCB concentration in fat + muscle) [45]. We calculated a partial fat basis for our samples based on the literature for percent fat found in deer (2.6 percent) and elk (2 percent) [46], resulting in concentrations of total PCBs based on a fat basis for rural deer of 0.42 ± 0.69 µg/kg fat and developed deer of 52.30 ± 347 µg/kg fat and a fat basis for rural elk of 0.17 ± 0.34 µg/kg fat and developed elk of 4.85 ± 6.55 µg/kg fat. The average of all locations was below FDA limits. One sample (a deer collected in Los Alamos township) had elevated levels of PCBs (63.3 µg/kg ww or approximately 2,434.61 µg/kg fat), which was approaching FDA levels; however, it is important to note that the fat basis for our calculation is only an estimate because fat content was inferred from literature and not measured directly in our samples. Within the omnivore and carnivore feeding guild, we found the mean concentration of total PCBs for black bears, coyotes, and mountain lions were 1.27, 2.86, and 4.01 ng/g ww respectively in muscle tissue. While direct comparisons can be difficult to make based on tissue type, we note similar concentrations between our investigation and studies conducted in brown bears (*Ursus arctos*), gray wolfs (*Canis lupus*), and bobcats, where Herceg Romanić noted concentration of total PCBs in adipose tissue of 2.88 ng/g fat in brown bears and 10.3 ng/g fat in gray wolfs in Croatia and Boyles found mean concentration of 562.97 ng/g fat in liver samples collected from bobcats in Illinois, United States [47,48].

We noted strong biomagnification of mid- to high-chlorinated PCBs in the carnivore feeding guild. When we examined biomagnification between PCB homologs prey (deer + elk) and predator (mountain lion) we noted strong biomagnification for pentachloro, nonachloro, and decachloro (BMFs ranging from 43.5 to 278.4) which suggests that apex predators experience disproportionately elevated contaminant burdens relative to their herbivore prey. This accumulation may increase risks of endocrine, immune, and reproductive effects [49–51] in top predators, which are already vulnerable due to their low abundance and large habitat requirements. The homolog-specific pattern indicates that heavier PCBs resist metabolic breakdown, allowing them to persist and move efficiently through the food web, highlighting the value of apex predators as indicators of broader ecosystem contamination. Biomagnification in top terrestrial predators has been estimated to be approximately 41 in grey wolves eating a lean diet and 81 for domestic dogs eating a lipid rich diet [52]. To our knowledge, PCB burdens and homolog-specific patterns in mountain lions have not previously been reported, making this study a novel contribution to understanding contaminant biomagnification in a terrestrial apex predator.

Although direct comparisons of TEQ muscle concentrations and adverse effects are lacking, concentrations observed here are at least one order of magnitude below adverse effect levels reported in other tissues. Sensitivity to TCDD toxicity exists both among and within taxa. For example, variable sensitivity within birds is primarily derived by amino acid differences in the AhR ligand binding domain, whereas overall differences in cytochrome P450 enzymes exist between mammals and birds. In the literature, it is reported that deleterious effects on hatching success in birds can occur at TEQ concentrations of approximately 1–11 ng/g ww in eggs [53]. In terrestrial mammals, the threshold tissue residue concentration of unacceptable risk is 32 ng/g lipid in the liver [54]. No TEQ effect levels for terrestrial reptiles were found. All TEQ min and TEQ max calculations across all taxa observed in this study were below 0.10 ng/g.

In our investigation, we were able to detect PCB homologs in all feeding guilds and locations sampled, which include rural, developed areas in New Mexico. Although PCBs have been officially banned from production in the United States, the contamination from decades of industrial use remains a concern. When we explored the differences between locations in which samples were collected, we noted that the samples collected in developed locations had significantly different distributions in total PCB concentrations (deer [ANOVA] and elk [ANOVA and NMDS] samples only) than those collected from rural locations. We also observed differences in the proportions of dioxin-like PCB observations in elk, where PCB-118 was the only congener observed in elk from rural locations. Although PCB-118 was the dominant dioxin-like PCB congener observed in elk from developed areas, these elk also contained PCB-105, PCB-156/157, and PCB-167. In general, PCBs in the air are those that contain low-chlorinated PCBs (5 chlorines or less), which could explain in part why PCB-118 (contains 5 chlorines) was the only dioxin-like congener observed in elk from rural locations [55]. From developed areas, we also observed high-chlorinated (6 or more chlorines) PCB congeners −156/157 and −167, which are less likely to be transported via air and could indicate a more direct exposure. However, PCB-105 is also a low-chlorinated PCB compound (contains 5 chlorines) but was not observed in rural elk. It has been previously demonstrated that PCB concentrations are higher in developed areas when compared with rural areas, and it might not come as a surprise that the higher mean PCB concentrations and greater diversity of dioxin-like PCBs in elk were observed in developed areas in our study [14,56–58].

The findings of this investigation provide a glimpse into PCB homolog distribution within muscle samples collected in New Mexico, United States. The findings suggest higher-trophic-level organisms bioaccumulate most PCBs homologs and dioxin-like PCB congeners. The continued monitoring of contaminates in the environment is of upmost importance because PCB contamination remains a concern for both environmental and human health. Furthermore, by demonstrating that PCBs tend to accumulate up the trophic levels, higher-trophic-level organisms might be at higher risk of adverse effects from these chemicals; this result could be especially true because the highest levels of PCBs congeners observed in the carnivore feeding guild contain some of the most toxic PCB congeners. Carnivore health is critical in maintaining a balanced ecosystem and provides an important indicator of broader ecosystem health. The findings of this investigation demonstrate the need for continued monitoring of PCBs in wildlife, which can help develop best management practices to reduce PCB levels in the environment.

## Supporting information

S1 Table PCB concentrations. (XLSX)

S2 Table Dioxin-like PCB concentrations. (XLSX)
